# The Efficacy and Safety of Hepatic Artery Infusion Chemotherapy Combined with Lenvatinib and Programmed Death (PD)-1 Inhibitors for Unresectable Intrahepatic Cholangiocarcinoma: A Retrospective Study

**DOI:** 10.3390/curroncol32020087

**Published:** 2025-02-04

**Authors:** Yingxiao Cai, Wu Wen, Yangshuo Xia, Renhua Wan

**Affiliations:** Department of General Surgery, The First Affiliated Hospital of Nanchang University, No. 17 Yongwaizheng Street, Donghu District, Nanchang 330006, China

**Keywords:** hepatic artery infusion chemotherapy, PD-1 inhibitors, overall survival, lenvatinib, unresectable intrahepatic cholangiocarcinoma

## Abstract

**Objectives:** Although systemic chemotherapy (SC) is the mainstay for treating unresectable intrahepatic cholangiocarcinoma (ICC), its efficacy is limited and it causes severe systemic side effects. This study focuses on evaluating the effectiveness and safety of hepatic arterial infusion chemotherapy (HAIC) in combination with lenvatinib plus programmed death-1 (PD-1) inhibitors (HLP), compared to SC in combination with lenvatinib plus PD-1 inhibitors (SCLP) for unresectable ICC. **Methods:** We analyzed patients initially diagnosed with unresectable ICC at our center between March 2021 and December 2023, classifying them into HLP and SCLP groups according to treatment regimen. This study assessed and compared overall survival (OS), progression-free survival (PFS), tumor response, and safety outcomes across the two treatment groups. **Results:** This study enrolled 53 subjects in total; 25 were treated with HLP and 28 with SCLP. The two groups showed well-matched baseline characteristics. The HLP group reported an extended median OS (12.8 vs. 11.0 months, *p* = 0.310) and a prolonged median PFS (8.8 vs. 6.4 months, *p* = 0.043), compared to the SCLP group. The HLP group had a better objective response rate (ORR) (52% vs. 25%, *p* = 0.043) and disease control rate (DCR) (96% vs. 78.6%, *p* = 0.104). Based on OS (*p* = 0.019) and PFS (*p* = 0.032) results, those without extrahepatic metastasis seemed to benefit more significantly from the HLP regimen than from the SCLP regimen. The HLP group experienced fewer grade 3–4 adverse events (AEs) than the SCLP group. **Conclusions:** The HLP regimen for unresectable ICC is an effective and safe strategy and is potentially better suited for patients without extrahepatic metastases.

## 1. Introduction

Intrahepatic cholangiocarcinoma (ICC) represents a condition formed by cancer cells in the bile ducts within the liver. Among all primary liver cancers, ICC is second only to that of hepatocellular carcinoma (HCC), comprising 10–15% of cases [1]. ICC occurrence has been showing a significant upward trend worldwide, with a notably higher rate in Asian populations compared to European populations [2,3,4]. ICC is highly aggressive and prone to both lymph node and extrahepatic metastasis by invading surrounding organs, tissues, and nerves, resulting in an unfavorable prognosis [5]. Achieving full surgical removal offers the greatest opportunity for extended survival among ICC patients [6]. Unfortunately, the majority of patients with ICC have reached an advanced local phase or presented with remote metastases upon diagnosis [7]. Advanced unresectable ICC has a poor outlook, with few available treatment options and a five-year survival rate that generally falls below 5% [8].

Systemic chemotherapy (SC) remains the primary treatment option for most cases of unresectable ICC. Among first-line therapies, the gemcitabine/cisplatin (GEMCIS) regimen has been established as the standard. The pivotal ABC-02 study demonstrated that the GEMCIS regimen significantly improved median overall survival (OS), achieving it at 11.7 versus 8.1 months, and increased median progression-free survival (PFS), reaching 5.0 versus 8.0 months, compared to gemcitabine monotherapy [9]. However, oxaliplatin is often preferred over cisplatin due to its lower risk of nephrotoxicity and the emergence of cisplatin resistance. A study reports that gemcitabine/oxaliplatin (GEMOX) has similar efficacy to GEMCIS and also reduces toxicity [10]. Additionally, a phase III trial demonstrated that a folinic acid, fluorouracil, and oxaliplatin (FOLFOX) regimen can significantly extend patients’ OS compared to active symptom control and has become the standard second-line therapy [11]. Despite the clinical benefits of SC for unresectable ICC, its efficacy remains limited, underscoring the urgent need for new treatment approaches.

Hepatic arterial infusion chemotherapy (HAIC) continuously delivers anticancer agents straight to the tumor’s blood supply through hepatic artery cannulation. This approach not only increases the drug concentration at the tumor site, allowing for longer drug–tumor cell interactions and enhanced targeting, but also reduces systemic toxicity [12,13]. Several studies have indicated that HAIC serves as a viable approach for managing unresectable ICC [14,15]. Developments in immunotherapy have made it possible to treat ICC with immune checkpoint inhibitors; typical examples include programmed death 1/L1 (PD-1/PD-L1) inhibitors [16,17]. Lenvatinib, an agent targeting multiple tyrosine kinases with anti-angiogenic properties, has demonstrated efficacy in individuals with unresectable ICC [18]. However, the efficacy of either PD-1 inhibitors or lenvatinib alone or together remains suboptimal [19]. Recent investigations have reported that a triple regimen combining HAIC, lenvatinib, and PD-1 inhibitors greatly improves outcomes in cases of unresectable HCC [20,21]. Despite these encouraging outcomes, reports on the use of this combination therapy in ICC remain limited.

There may be a combined effect among HAIC, lenvatinib, and PD-1 inhibitors. Defining the advantages and possible adverse effects of this treatment combination is crucial for refining therapeutic approaches and enhancing patient outcomes. This article presents a study evaluating the effectiveness and safety of HAIC, lenvatinib, and PD-1 inhibitors (HLP) as a combination approach in managing unresectable ICC, comparing clinical outcomes with those in patients undergoing SC, lenvatinib, and PD-1 inhibitors (SCLP) in a similar center and period.

## 2. Materials and Methods

### 2.1. Study Subject and Selection Criteria

This retrospective study, carried out at the First Affiliated Hospital of Nanchang University from March 2021 to December 2023, involved 53 patients receiving either HLP or SCLP treatment as first-line therapy. Participants qualified for inclusion in this study provided they fulfilled the specific criteria outlined below: (1) they were initially diagnosed with ICC confirmed by both pathology and imaging, and their disease had been assessed as unresectable by two experienced hepatobiliary surgeons. Unresectable cases include tumors closely adjacent to major blood vessels, making R0 resection unachievable; those with vascular invasion, lymph node metastasis, or distant metastasis; and cases where the remaining liver volume after resection does not meet the criteria of the safe liver resection decision-making system; (2) they were aged between 18 and 78 years; (3) they had undergone at least one cycle of HAIC or SC treatment, both alongside lenvatinib and a type of PD-1 inhibitors; (4) they had an Eastern Cooperative Oncology Group (ECOG) performance status score of either 0 or 1; and (5) they presented with a minimum of evaluable target lesion detectable through imaging as defined by the Response Evaluation Criteria in Solid Tumors (RECIST) 1.1 criteria. The primary reasons for exclusion are listed below: (1) liver function evaluation criteria (Child–Pugh) class C; (2) history of other malignancies; (3) anticipated survival time of under 3 months; (4) incomplete clinical data; or (5) lost to follow-up.

### 2.2. Treatments

Before undergoing HAIC treatment, it is essential to ensure that patients have adequate liver function reserve. For patients with significant obstructive jaundice due to bile duct obstruction, percutaneous transhepatic catheter drainage(PTCD) drainage should be performed before HAIC to reduce bilirubin levels to less than three times the normal value. At our center, under local anesthesia, the Seldinger technique is used to puncture the right femoral artery, and tumor-feeding vessels are visualized using digital subtraction angiography. A microcatheter is then super-selected into the tumor-feeding artery. Finally, GEMOX chemotherapy is infused on the ward, as follows: gemcitabine (1000 mg/m^2^ over 30 min) on the first day, followed by oxaliplatin (85 mg/m^2^ over 2 h) on the same day. HAIC was administered every three weeks, with patients in this study receiving between one and eight treatment cycles.

Two regimens were used for SC. For the GEMCIS, every cycle involved adminis tering cisplatin (25 mg/m^2^) and gemcitabine (1000 mg/m^2^), given on both the first and eighth days. Another GEMOX cycle included oxaliplatin (85 mg/m^2^) on the first day, with gemcitabine (1000 mg/m^2^) given according to the same schedule. Both regimens were administered every three weeks. Dose adjustments, such as a reduction in or omission of chemotherapy on day 8, were considered if the patient exhibited intolerance, continuing until disease advancement.

PD-1 inhibitors were given intravenously for 1 to 3 days following HAIC or SC. The options included 200 mg of camrelizumab, tislelizumab, or sintilimab, and 240 mg of toripalimab, administered on a triweekly basis. Lenvatinib was administered orally, at a dosage 12 mg or 8 mg daily depending on whether the patient weighed ≥ 60 kg or <60 kg, respectively, with dosage adjustments based on the severity of adverse reactions. If the treatment showed effectiveness, a multidisciplinary team would meet to determine whether to move forward with conversion surgery for resection or to continue treatment involving lenvatinib and PD-1 inhibitors.

### 2.3. Data Acquisition and Assessment

Clinical data for all patients were collected from our center’s electronic medical record system, including demographic indicators (e.g., age, gender, HBV, ECOG performance status, Child–Pugh classification, etc.), tumor-related information (e.g., largest tumor dimension, tumor numbers, vascular invasion, lymph node metastasis, extrahepatic metastasis, tumor TNM stage, number of treatment cycles, etc.), and relevant laboratory parameters (e.g., carbohydrate antigen 19-9 (CA19-9), carcinoembryonic antigen (CEA), aspartate aminotransferase (AST), alanine aminotransferase (ALT), albumin (ALB), alkaline phosphatase (ALP), gamma-glutamyl transferase (GGT), etc.). Before the first treatment within 1–2 weeks, patients underwent laboratory tests and imaging studies to evaluate the target lesion. Thereafter, laboratory evaluations were performed ahead of each treatment to monitor treatment-related complications. During the treatment period, tumor response underwent assessment every 6 to 8 weeks following RECIST 1.1 and modified RECIST (mRECIST) criteria, with the best response recorded by at least two specialized radiologists without information on the patient’s therapeutic plans or results. The study continued until 1 July 2024.

Progression-free survival (PFS), as the primary endpoint, refers to the period starting with the first HAIC or SC treatment, extending until tumor progression, death due to the tumor, or the most recent follow-up, whichever happens first. Overall survival (OS) is defined as the timeframe beginning with HAIC or SC treatment initiation and continuing until death from tumor-related causes or the most recent follow-up. Tumor response indicators include complete response (CR), partial response (PR), stable disease (SD), and progressive disease (PD). The overall response rate (ORR) comprises CR and PR, while the disease control rate (DCR) encompasses CR, PR, and SD. The assessment of AEs was conducted using the Common Terminology Criteria for Adverse Events (CTCAE) version 5.0.

### 2.4. Data Analysis

Categorical variables across the patients’ baseline profile were analyzed with the chi-square test or Fisher’s exact test; while measurements following normal distribution were reported with the *t*-test as mean ± SD, for data that were not normally distributed, the median (IQR) was presented using the rank-sum test. The Kaplan–Meier method produced survival curves for PFS and OS, with differences between them evaluated using the log-rank test. Factors potentially influencing patients’ prognosis were examined through univariate analyses, and those with a *p*-value < 0.1 were then incorporated into multiple regression analyses. A two-sided *p*-value < 0.05 demonstrates a statistical difference. All statistical analyses were conducted with RStudio (version 4.3.1, R Foundation for Statistical Computing, Vienna, Austria) and SPSS (version 26, IBM Corp., Armonk, NY, USA).

## 3. Results

### 3.1. Patient Characteristics

**Figure 1:**
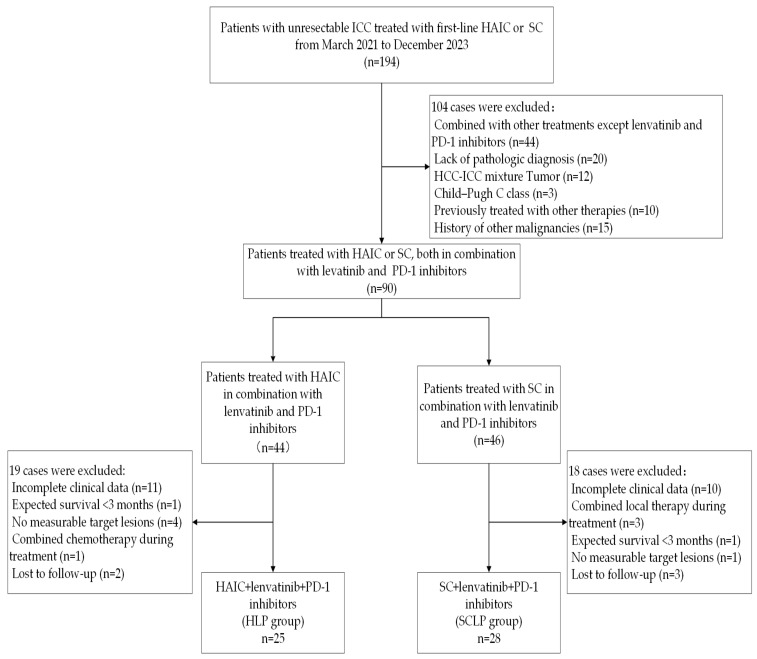
Flowchart depicting the experimental design.

We ultimately included 53 patients with unresectable ICC, with 25 treated with HLP and 28 treated with SCLP (Figure 1). The HLP and SCLP groups had comparable baselines; see Table 1 for details. Patients in the HLP group underwent one to eight cycles, averaging three, while those in the SCLP group underwent one to nine cycles, averaging four. The average largest tumor diameter in the HLP group was 8.13 cm, with 52% having multiple tumors, and 84% in TMN stages III–IV. In contrast, the average largest tumor diameter in the SCLP group was 7.1 cm, with 67.9% having multiple tumors and 85.7% in TMN stages III–IV. Most patients presented with high-burden tumors and later TNM stages, consistent with the theme of unresectability.

### 3.2. Survival

**Figure 2:**
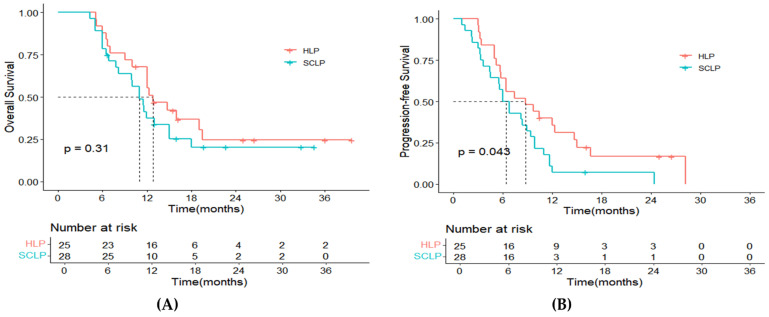
Kaplan–Meier plots for overall survival (**A**) and progression-free survival (**B**) for all patients. Notes: HLP, hepatic artery infusion chemotherapy with lenvatinib and PD-1 inhibitors; SCLP, systemic chemotherapy with lenvatinib and PD-1 inhibitors.

**Figure 3:**
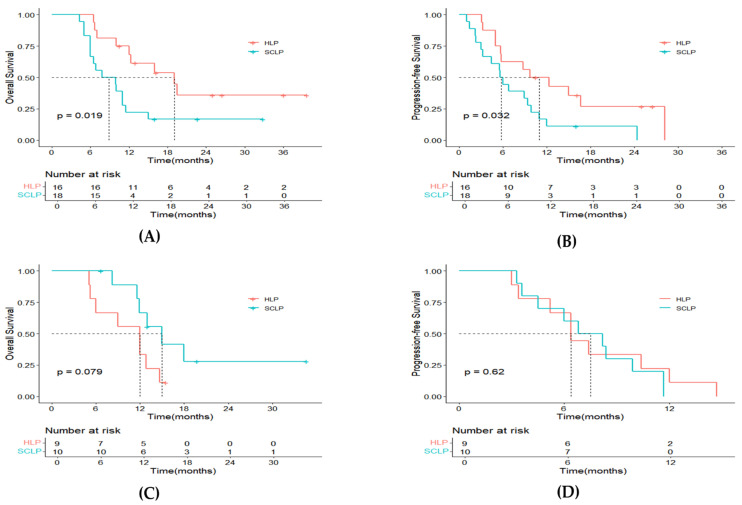
Kaplan–Meier plots for overall survival (**A**) and progression-free survival (**B**) for patients without extrahepatic metastasis and overall survival (**C**) and progression-free survival (**D**) for patients with extrahepatic metastasis. Notes: HLP, hepatic artery infusion chemotherapy with lenvatinib and PD-1 inhibitors; SCLP, systemic chemotherapy with lenvatinib and PD-1 inhibitors.

The follow-up duration medians were 12.3 months (range: 5.1–39.7) and 11.0 months (range: 4.3–34.6) for the two treatment groups, respectively. Up to the latest follow-up on 1 July 2024, disease progression occurred in 21 patients (84%) from the HLP group, with 17 (68%) deaths. In the SCLP group, 27 patients (96%) had tumor progression, and 21 (75%) had passed away. The median OS for the HLP group was 12.8 months (95% CI: 8.7–16.9), compared to 11.0 months (95% CI: 8.5–13.5) for the SCLP group (*p* = 0.310, Figure 2A). For PFS, the HLP group showed a median of 8.8 months (95% CI: 3.4–14.2), notably exceeding the 6.4 months (95% CI: 4.4–7.6) observed for the SCLP group (*p* = 0.043, Figure 2B). By analyzing patients without extrahepatic metastasis as a separate subgroup, we found that those in the HLP group showed significantly superior results in not only OS (*p* = 0.019, Figure 3A) but also PFS (*p* = 0.032, Figure 3B). However, for patients with extrahepatic metastases, the OS (*p* = 0.079, Figure 3C) and PFS (*p* = 0.620, Figure 3D) in the HLP group showed no significant differences compared to those in the SCLP group.

### 3.3. Tumor Response

**Figure 4:**
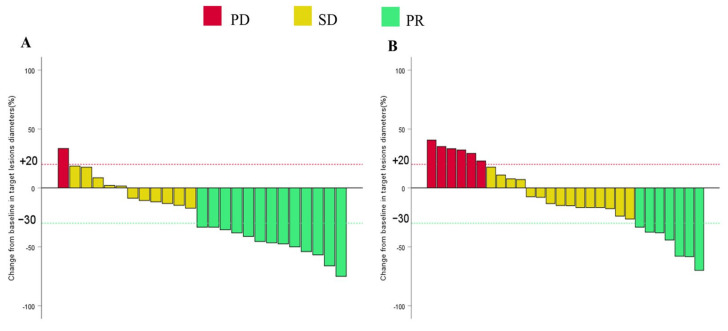
Waterfall plots for maximum percentage change in the sum of the sizes of the target lesions from baseline as assessed per RECIST 1.1 in the HLP group (**A**) and the SCLP group (**B**). Notes: PR, partial response; SD, stable disease; PD, progressive disease.

An elevated ORR (52% vs. 25%, *p* = 0.043, based on RECIST 1.1; 68% vs. 32.1%, *p* = 0.009, based on mRECIST) and DCR (96% vs. 78.6%, *p* = 0.104) were observed in the HLP group compared to the SCLP group (Table 2). Moreover, two patients (8.0%) from the HLP group and one patient (3.6%) from the SCLP group successfully underwent R0 resection after treatment. Their postoperative pathology confirmed that one patient achieved pathological complete response (pCR), while another patient exhibited extensive tumor necrosis. As shown in Figure 4, waterfall plots depicting variations in target lesion sizes relative to baseline, assessed according to RECIST 1.1 standards, showed a decrease in lesion size in 19 cases (76%) for the HLP group and in 18 cases (64%) for the SCLP group.

### 3.4. Prognostic Factors Analyses

The results of the analysis of all prognostic factors that may influence patients’ OS and PFS are shown in Table 3. Multivariate analyses found that ALB < 35 (*p* = 0.045) and TNM stages III-IV (*p* = 0.014) acted as independent negative predictors of OS, while the completion of ≥four treatment cycles of treatment (*p* = 0.014) emerged as a favorable factor. Regarding PFS, an ECOG performance status score of one (*p* = 0.023) was recognized as an independent risk factor, while HLP therapy (*p* = 0.011) and completing ≥ four treatment cycles (*p* = 0.004) proved to be significantly distinct advantageous factors.

## 4. Safety

Adverse events (AEs) underwent comprehensive evaluation in both groups, with findings summarized in Table 4. Overall, AEs occurred less frequently in the HLP group compared to the SCLP group. The predominant AEs were elevated AST (12/25, 48%), elevated ALT (10/25, 40%), abdominal pain or bloating (10/25, 40%), vomiting (9/25, 36%), dysuria (7/25, 28%), hand-foot syndrome (7/25, 28%), hypoproteinemia (7/25, 28%), elevated blood pressure (7/25, 28%), and leukopenia (5/25, 20%) in the HLP group. In contrast, the SCLP group most frequently experienced nausea and vomiting (20/28, 71.4%), leukopenia (15/28, 53.6%), anemia (13/28, 46.4%), fatigue (11/28, 39.3%), elevated AST (10/28, 35.7%), elevated ALT (8/28, 28.6%), and thrombocytopenia (7/28, 25%). Notably, fatigue (11/28, 39.3% vs. 3/25, 12%, *p* = 0.025), vomiting (20/28, 71.4% vs. 9/25, 36%, *p* = 0.01), leukopenia (15/28, 53.6% vs. 5/25, 20%, *p* = 0.012), and anemia (13/28, 46.4% vs. 3/25, 12%, *p* = 0.006) AEs were more prevalent in the SCLP group. Additionally, grade 3–4 AEs of vomiting (8/28, 28.6% vs. 1/25, 4%, *p* = 0.044), leukopenia (6/28, 21.4% vs. 0/25, 0%, *p* = 0.024), and anemia (6/28, 21.4% vs. 0/25, 0%, *p* = 0.024) AEs had a higher incidence in the SCLP group. Most AEs can be controlled after symptomatic treatment. No deaths associated with AEs were observed among any patients.

## 5. Discussion

Based on the findings of our study, the HLP regimen indeed provided higher tumor response rates and longer survival for unresectable ICC, compared to SCLP treatment. This suggests that HLP could be a more effective treatment option for unresectable ICC. Prior investigations have indicated that HAIC benefits patients suffering from unresectable advanced ICC, even demonstrating superior tumor control compared to SC [22,23,24,25]. Regarding OS, although our study did not achieve statistical significance, the survival curves revealed that the survival rate in the HLP group was higher than in the SCLP group at most time points, suggesting a potential survival advantage for the HLP group. Larger-sample analyses are needed in the future to confirm this conclusion. Additionally, among patients without extrahepatic metastasis, those receiving HLP treatment had significantly higher OS and PFS than those receiving SCLP, indicating that the HLP regimen may be more suitable for ICC patients without extrahepatic metastasis. Our findings provide clinical evidence for using the HLP regimen in this specific patient population.

Compared to HCC, ICC is more malignant and has a more insidious onset, with over 30% of patients losing the opportunity for curative surgery upon confirmation of the diagnosis [26]. For unresectable ICC, gemcitabine-based systemic chemotherapy has been the first-line treatment over the past decades [9,10]. Advances in next-generation sequencing technology and widespread precision medicine have led to the introduction of immunotherapy and targeted treatments, reshaping the traditional chemotherapy paradigm for ICC. Two multicenter phase III clinical trials reported that the combination of the PD-L1 inhibitor durvalumab with GEMCIS chemotherapy and the PD-1 inhibitor pembrolizumab with GEMCIS chemotherapy both significantly prolonged median OS compared to GEMCIS chemotherapy alone, and have become new first-line treatment options [27,28]. Notably, the median OS of the HLP group in this study is similar to the results of the aforementioned two studies. Lenvatinib has been proven to be non-inferior to sorafenib in OS for untreated advanced HCC [29]. Reports on lenvatinib monotherapy for ICC are limited. The results of a phase II study conducted in 2021 evaluating toripalimab combined with lenvatinib as a first-line treatment for advanced ICC indicated that, among 31 participants, the ORR impressively reached 32.3%, with the DCR at 74.2%, and with the 6-month OS rate achieving a notable 87.1%. Moreover, two patients successfully underwent surgical resection after treatment [30]. Additionally, two studies evaluating the treatment of lenvatinib plus PD-1 inhibitors for chemotherapy-refractory or chemotherapy-refusing ICC reported median OS of 11.4 and 14.3 months, with median PFS of 5.9 and 5.83 months, respectively [31,32].

Locoregional therapy represents a viable therapeutic approach for patients with unresectable ICC. It not only facilitates control of the primary tumor burden, thereby delaying disease progression, but also serves as a conversion strategy to achieve tumor downstaging and subsequent resection [33,34,35]. Currently, the primary modalities of locoregional therapy include hepatic arterial infusion chemotherapy (HAIC), transarterial chemoembolization (TACE), radiofrequency ablation (RFA), and transarterial radioembolization (TARE), with multiple studies confirming their efficacy in the management of unresectable ICC [15,36,37,38]. Nevertheless, evidence suggests that the outcomes of locoregional therapy alone remain suboptimal, particularly in patients with distant metastases, where its clinical benefits are limited. As a result, there is increasing interest in exploring combination treatment strategies that integrate locoregional and systemic therapies to evaluate their potential in improving the prognosis of patients with unresectable ICC. A prospective study evaluated the efficacy of drug-eluting beads transarterial chemoembolization(DEB-TACE) combined with GEMCIS chemotherapy for unresectable ICC, demonstrating significantly better OS (33.7 vs. 12.6 months) and PFS (31.9 vs. 10.1 months) compared to GEMCIS chemotherapy alone [39]. Similarly, a retrospective analysis revealed that HAIC combined with systemic chemotherapy (SC) significantly improved the median OS (30.8 months vs. 18.4 months) and achieved a higher PR rate compared to SC alone (59% vs. 39%) [40]. Building on these findings, a single-arm study involving 36 patients with unresectable biliary tract cancer treated with HAIC and PD-1 inhibitors reported a median OS of 8.8 months and a median PFS of 3.7 months [41]. Moreover, Wang et al. conducted a retrospective study and found that combining locoregional therapy with toripalimab and lenvatinib resulted in longer OS and PFS, compared to the toripalimab and lenvatinib group alone, for treating unresectable biliary tract cancer [42]. Most recently, a study demonstrated the superior efficacy of HAIC combined with intravenous gemcitabine chemotherapy, lenvatinib, and PD-1 inhibitors in managing large, unresectable ICC. Among 21 patients, the median OS reached 19.5 months, the median PFS was 6.0 months, and the DCR and ORR were 76.1% and 52.3%, respectively [43]. These findings collectively highlight the potential of combination strategies in improving outcomes for patients with unresectable ICC.

The improved remission rates and extended survival observed with the HLP regimen (HAIC, lenvatinib, and PD-1 inhibitors) are likely due to the complementary actions of these therapies. HAIC maintains high intratumoral chemotherapy concentrations, reducing tumor burden and inducing immunogenic cell death (ICD), which enhances PD-1 inhibitor efficacy by providing a richer antigen pool for T-cell activation [44]. Lenvatinib suppresses tumors by blocking angiogenesis, reshaping the tumor microenvironment (TME), and improving immune cell infiltration and vascular structure, which increases PD-1 inhibitor responsiveness and reduces chemoresistance [45,46]. This triple therapy effectively modifies the hypoxic TME, boosts drug delivery [47], and holds significant potential as a therapeutic strategy for ICC patients.

The incidence of AEs is a crucial indicator for evaluating treatment regimens. In this research, no patients encountered fatal AEs. The most frequent AEs observed in both groups were hepatic dysfunction, gastrointestinal symptoms, and myelosuppression. This phenomenon may be ascribed to the concentration of primary lesions within the liver; while both therapeutic approaches are effective in eradicating tumor cells, they also compromise normal hepatocytes, resulting in hepatic dysfunction. Furthermore, chemotherapeutic agents, disseminated via systemic circulation, predispose patients to gastrointestinal disturbances and bone marrow suppression. Notably, our analysis revealed a significantly higher incidence of grade 3–4 adverse reactions, including leukopenia, vomiting, and anemia, within the SCLP group. Although individuals in the HLP group encountered certain grade 3–4 AEs, these were manageable, safe, and non-fatal. Additionally, our study found that advanced TNM staging and lower albumin levels were significant risk factors for OS, while tumor multiplicity and higher ECOG performance status scores were linked to worse PFS. Notably, receiving more than four treatment cycles appeared to improve a patient’s survival prognosis. These findings could provide valuable insights for clinical treatment planning.

This study has a few shortcomings. This study, as it was conducted at a single center, will unavoidably have selection bias. Secondly, the limited sample size and brief follow-up duration are additional constraints that will limit the statistical analysis effectiveness and the applicability of the results. Moreover, the lack of a standardized first-line treatment as a control limits the study’s comprehensiveness. Despite these limitations, the findings indicate that the HLP regimen may offer therapeutic benefits in managing ICC. This presents a potential avenue for future treatment strategies. However, these results should be interpreted cautiously, given the study’s limitations. Future research should focus on validating these outcomes through large-scale, randomized clinical studies, helping to clarify the HLP regimen’s function in managing ICC and providing stronger evidence for clinical practice.

## 6. Conclusions

In summary, our study demonstrated that the HLP triple regimen is an effective, safe, and manageable treatment strategy for unresectable ICC, particularly in patients without extrahepatic metastases, offering significant survival benefits for this subgroup.

## Figures and Tables

**Table 1:** The baseline characteristics of all patients (n = 53).

Characteristic	HLP Group n = 25	SCLP Group n = 28	*p*-Value
Gender, n (%)			0.610
Male	16 (64%)	16 (57%)	
Female	9 (16%)	12 (43%)	
Age, median (IQR), years	63 (53.5–66)	58.5 (52.75–65)	0.574
HBV, n (%)			0.365
postive	12 (48.0%)	10 (35.7%)	
negative	13 (52.0%)	18 (64.3%)	
ECOG performance status, n (%)			0.963
0	18 (72.0%)	20 (71.4%)	
1	7 (28.0%)	8 (28.6%)	
Child–Pugh class, n (%)			0.355
A	24 (96.0%)	24 (85.7%)	
B	1 (4.0%)	4 (14.3%)	
Liver cirrhosis, n (%)			0.243
Yes	10 (40.0%)	7 (25%)	
No	15 (60.0%)	21 (75%)	
Cycle times, median (IQR)	3 (2–4)	3 (2–5)	0.270
Extrahepatic metastasis, n (%)			0.983
Yes	9 (36.0%)	10 (35.7%)	
No	16 (64.0%)	18 (64.3%)	
Lymph node metastasis, n (%)			0.706
Yes	19 (76.0%)	20 (71.4%)	
No	6 (24.0%)	8 (28.6%)	
Vascular invasion, n (%)			0.135
Yes	9 (36.0%)	5 (17.9%)	
No	16 (64.0%)	23 (82.1%)	
Tumor numbers, n (%)			0.239
Single	12 (48.0%)	9 (32.1%)	
Multiple	13 (52.0%)	19 (67.9%)	
TNM stage, n (%)			1.000
I–II	4 (16.0%)	4 (14.3%)	
III–IV	21 (84.0%)	24 (85.7%)	
Largest tumor dimension, mean ± SD, cm	8.13 ± 3.59	7.10 ± 3.52	0.323
CA19-9, median (IQR), U/ml	165 (30.975–901)	417.65 (23.275–2554.65)	0.715
CEA, median (IQR), U/m	4.45 (2.335–15.455)	3.56 (1.703–8.01)	0.340
AST, median (IQR), U/L	38.4 (28.15–50.25)	35.5 (22.83–44.15)	0.190
ALT, median (IQR), U/L	25.7 (17.2–45.5)	23.8 (20.0–37.14)	0.831
ALB, mean ± SD, g/L	38.42 ± 3.494	38.812 ± 4.097	0.712
GGT, median (IQR), U/L	147 (70.50–259.55)	78.67 (53.95–183.75)	0.113
ALP, median (IQR), U/L	164.1 (93.10–240.20)	132.77 (97.80–174.91)	0.412

Notes: HLP, hepatic artery infusion chemotherapy with Lenvatinib and PD-1 inhibitors; SCLP, systemic chemotherapy with lenvatinib and PD-1 inhibitors; ECOG, Eastern Cooperative Oncology Group; TNM, tumor–node–metastasis; CA19-9, carbohydrate antigen 19-9; CEA, carcinoembryonic antigen; AST, aspartate aminotransferase; ALT, alanine aminotransferase; ALB, albumin; GGT, gamma-glutamyl transferase; ALP, alkaline phosphatase.

**Table 2:** Tumor response and surgery rate between groups.

Tumor Response	RECIST1.1			mRECIST		
	HLP Group n, (%)	SCLP Group n, (%)	*p*-Value	HLP Group n, (%)	SCLP Group n, (%)	*p*-Value
CR	0 (0)	0 (0)	-	1 (4.0%)	0 (0)	-
PR	13 (52.0%)	7 (25.0%)	-	16 (64.0%)	9 (32.1%)	-
SD	11 (48.0%)	15 (53.6%)	-	9 (36.0%)	13 (46.4%)	-
PD	1 (4.0%)	6 (21.4%)	-	1 (4.0%)	6 (21.4%)	-
ORR	13 (44.0%)	7 (25.0%)	0.043	17 (68.0%)	9 (32.1%)	0.009
DCR	24 (96.0%)	22 (78.6%)	0.143	24 (96.0%)	22 (78.6%)	0.143
surgery after treatment	2 (8.0%)	1 (3.6%)	0.919	2 (8.0%)	1 (3.6%)	0.919

Notes: HLP, hepatic artery infusion chemotherapy with lenvatinib and PD-1 inhibitors; SCLP, systemic chemotherapy with lenvatinib and PD-1 inhibitors; CR, complete response; PR, partial response; SD, stable disease; PD, progressive disease; ORR, objective response rate; DCR, disease control rate. ORR = CR + PR; DCR = CR + PR + SD.

**Table 3:** Univariate and multivariate Cox regression analyses of risk factors for overall survival and progression-free survival.

Variables	Overall Survival				Progression-Free Survival			
	Univariate		Multivariate		Univariate		Multivariate	
	HR95%CI	*p*-Vaule	HR95%CI	*p*-Vaule	HR95%CI	*p*-Vaule	HR95%CI	*p*-Vaule
Treatment regimen (HLP)	0.721 (0.379–1.369)	0.317			0.551 (0.305–0.994)	0.048	0.434 (0.228–0.828)	0.011
Gender (Male)	1.253 (0.647–2.428)	0.504			1.506 (0.831–2.730)	0.177		
Age (≥60)	1.043 (0.549–1.983)	0.898			1.233 (0.682–2.227)	0.488		
HBV (Postive)	1.154 (0.607–2.192)	0.663			0.782 (0.432–1.415)	0.416		
ECOG performance status (1)	1.561 (0.785–3.107)	0.204			1.810 (0.965–3.396)	0.065	2.212 (1.1.108–4.057)	0.023
Child–Pugh (A)	0.488 (0.210–1.185)	0.113			0.755 (0.318–1.793)	0.525		
Liver cirrhosis (Yes)	0.998 (0.509–1.955)	0.994			0.860 (0.464–1.595)	0.632		
Lymph node metastasis (Yes)	1.817 (0.823–4.009)	0.139			1.513 (0.776–2.953)	0.225		
Extrahepatic metastasis (Yes)	1.009 (0.519–1.960)	0.979			1.540 (0.840–2.824)	0.163		
Vascular invasion (Yes)	1.109 (0.537–2.288)	0.780			0.937 (0.482–1.818)	0.847		
Tumor numbers (Multiple)	1.485 (0.776–2.878)	0.242			2.001 (1.085–3.690)	0.026	1.841 (0.985–3.442)	0.056
Largest tumor dimension (≥10)	1.555 (0.779–3.105)	0.210			1.198 (0.617–2.326)	0.593		
TNM stage (III-IV)	2.448 (0.870–7.115)	0.089	2.257 (0.546–0.935)	0.014	2.395 (0.985–5.824)	0.054	1.841 (0.985–3.442)	0.266
Cycle times (≥4)	0.393 (0.199–0.778)	0.007	0.434 (0.216–0.873)	0.019	0.463 (0.257–0.832)	0.010	0.383 (0.201–0.732)	0.004
CA19-9 (≥100)	1.338 (0.723–2.664)	0.324			0.914 (0.510–1.636)	0.761		
GGT (≥60)	3.176 (1.236–8.161)	0.016	2.305 (0.868–6.120)	0.094	1.217 (0.630–2.349)	0.558		
ALB (<35)	2.694 (1.162–6.245)	0.021	2.415 (1.018–5.728)	0.045	1.959 (0.847–4.530)	0.116		

Notes: HLP, hepatic artery infusion chemotherapy with lenvatinib and PD-1 inhibitors; ECOG, Eastern Cooperative Oncology Group; TNM, tumor–node–metastasis; CA19–9, carbohydrate antigen 19-9; ALB, albumin; GGT, gamma-glutamyl transferase.

**Table 4:** Summary of adverse events between groups.

Adverse Events	Any Grade			Grade 3–4		
	HLP Group	SCLP Group	*p*-Value	HLP Group	SCLP Group	*p*-Value
	n = 25	n = 28		n = 25	n = 28	
**Treatment-related AEs, n (%)**						
Fatigue	3 (12.0%)	11 (39.3%)	0.025	0	0	-
Fever	4 (16.0%)	4 (14.3%)	1.000	1 (4.0%)	0	0.954
Vomiting	9 (36.0%)	20 (71.4%)	0.010	1 (4.0%)	8 (28.6%)	0.044
Abdominal pain	10 (40.0%)	5 (18.9%)	0.074	2 (8.0%)	0	0.422
Rash	5 (20.0%)	7 (25.0%)	0.664	0	0	-
Hand-foot syndrome	7 (28.0%)	8 (28.6%)	0.963	1 (4.0%)	1 (3.6%)	1.000
Diarrhea or constipation	1 (4.0%)	2 (7.1%)	1.000	0	0	-
Loss of appetite	2 (8.0%)	7 (25.0%)	0.148	0	0	-
Elevated blood pressure	7 (28.0%)	5 (18.9%)	0.378	1 (4.0%)	0	0.954
Canker sore	3 (12.0%)	5 (18.9%)	0.833	0	0	-
Dysuria	7 (28.0%)	2 (7.1%)	0.098	0	0	-
**Laboratory-related AEs, n (%)**						
**Elevated AST**	12 (48.0%)	10 (35.7%)	0.365	1 (4.0%)	0	0.954
Elevated ALT	10 (40.0%)	8 (28.6%)	0.380	0	1 (3.6%)	0.954
Elevated total bilirubin	5 (20.0%)	4 (14.3%)	0.719	0	0	-
Hypothyroidism	4 (16.0%)	3 (10.7%)	0.694	0	0	-
Hypokalemia	1 (4.0%)	0	0.954	0	0	-
Elevated creatinine	0	1 (3.6%)	1.000	0	0	-
Hypoproteinemia	7 (28.0%)	5 (18.9%)	0.378	1 (4.0%)	0	0.954
Hyperalgesia	3 (12.0%)	0	0.196	0	0	-
Anemic	3 (12.0%)	13 (46.4%)	0.006	0	6 (21.4%)	0.024
Thrombocytopenia	2 (8.0%)	7 (25%)	0.148	0	1 (3.6%)	0.954
Leukopenia	5 (20.0%)	15 (53.6%)	0.012	0	6 (21.4%)	0.024
Neutropenia	3 (12.0%)	8 (28.6%)	0.138	0	2 (7.1%)	0.492
Proteinuria	1 (4.0%)	0	0.954	0	0	-

Notes: HLP, hepatic artery infusion chemotherapy with lenvatinib and PD-1 inhibitors; SCLP, systemic chemotherapy with lenvatinib and PD-1 inhibitors; AST, aspartate aminotransferase; ALT, alanine aminotransferase.

## Data Availability

The original contributions presented in this study are included in the article. Further inquiries can be directed to the corresponding author(s).

## References

[1] Siegel R.L., Miller K.D., Fuchs H.E., Jemal A. (2022). Cancer statistics, 2022. CA Cancer J. Clin..

[2] Banales J.M., Cardinale V., Carpino G., Marzioni M., Andersen J.B., Invernizzi P., Lind G.E., Folseraas T., Forbes S.J., Fouassier L. (2016). Expert consensus document: Cholangiocarcinoma: Current knowledge and future perspectives consensus statement from the European Network for the Study of Cholangiocarcinoma (ENS-CCA). Nat. Rev. Gastroenterol. Hepatol..

[3] Valle J.W., Borbath I., Khan S.A., Huguet F., Gruenberger T., Arnold D., Committee E.G. (2016). Biliary cancer: ESMO Clinical Practice Guidelines for diagnosis, treatment and follow-up. Ann. Oncol..

[4] Saha S.K., Zhu A.X., Fuchs C.S., Brooks G.A. (2016). Forty-Year Trends in Cholangiocarcinoma Incidence in the U.S.: Intrahepatic Disease on the Rise. Oncologist.

[5] Esnaola N.F., Meyer J.E., Karachristos A., Maranki J.L., Camp E.R., Denlinger C.S. (2016). Evaluation and management of intrahepatic and extrahepatic cholangiocarcinoma. Cancer.

[6] Lang H., Sotiropoulos G.C., Sgourakis G., Schmitz K.J., Paul A., Hilgard P., Zopf T., Trarbach T., Malago M., Baba H.A. (2009). Operations for intrahepatic cholangiocarcinoma: Single-institution experience of 158 patients. J. Am. Coll. Surg..

[7] Nathan H., Aloia T.A., Vauthey J.N., Abdalla E.K., Zhu A.X., Schulick R.D., Choti M.A., Pawlik T.M. (2009). A proposed staging system for intrahepatic cholangiocarcinoma. Ann. Surg. Oncol..

[8] Endo I., Gonen M., Yopp A.C., Dalal K.M., Zhou Q., Klimstra D., D’Angelica M., DeMatteo R.P., Fong Y., Schwartz L. (2008). Intrahepatic cholangiocarcinoma: Rising frequency, improved survival, and determinants of outcome after resection. Ann. Surg..

[9] Valle J., Wasan H., Palmer D.H., Cunningham D., Anthoney A., Maraveyas A., Madhusudan S., Iveson T., Hughes S., Pereira S.P. (2010). Cisplatin plus gemcitabine versus gemcitabine for biliary tract cancer. N. Engl. J. Med..

[10] Fiteni F., Nguyen T., Vernerey D., Paillard M.J., Kim S., Demarchi M., Fein F., Borg C., Bonnetain F., Pivot X. (2014). Cisplatin/gemcitabine or oxaliplatin/gemcitabine in the treatment of advanced biliary tract cancer: A systematic review. Cancer Med..

[11] Lamarca A., Palmer D.H., Wasan H.S., Ross P.J., Ma Y.T., Arora A., Falk S., Gillmore R., Wadsley J., Patel K. (2021). Second-line FOLFOX chemotherapy versus active symptom control for advanced biliary tract cancer (ABC-06): A phase 3, open-label, randomised, controlled trial. Lancet Oncol..

[12] Obi S., Sato S., Kawai T. (2015). Current Status of Hepatic Arterial Infusion Chemotherapy. Liver Cancer.

[13] Kudo M. (2012). Treatment of advanced hepatocellular carcinoma with emphasis on hepatic arterial infusion chemotherapy and molecular targeted therapy. Liver Cancer.

[14] Jarnagin W.R., Schwartz L.H., Gultekin D.H., Gonen M., Haviland D., Shia J., D’Angelica M., Fong Y., DeMatteo R., Tse A. (2009). Regional chemotherapy for unresectable primary liver cancer: Results of a phase II clinical trial and assessment of DCE-MRI as a biomarker of survival. Ann. Oncol..

[15] Boehm L.M., Jayakrishnan T.T., Miura J.T., Zacharias A.J., Johnston F.M., Turaga K.K., Gamblin T.C. (2015). Comparative effectiveness of hepatic artery based therapies for unresectable intrahepatic cholangiocarcinoma. J. Surg. Oncol..

[16] Piha-Paul S.A., Oh D.Y., Ueno M., Malka D., Chung H.C., Nagrial A., Kelley R.K., Ros W., Italiano A., Nakagawa K. (2020). Efficacy and safety of pembrolizumab for the treatment of advanced biliary cancer: Results from the KEYNOTE-158 and KEYNOTE-028 studies. Int. J. Cancer.

[17] Kim R.D., Chung V., Alese O.B., El-Rayes B.F., Li D., Al-Toubah T.E., Schell M.J., Zhou J.M., Mahipal A., Kim B.H. (2020). A Phase 2 Multi-institutional Study of Nivolumab for Patients with Advanced Refractory Biliary Tract Cancer. JAMA Oncol..

[18] Ueno M., Ikeda M., Sasaki T., Nagashima F., Mizuno N., Shimizu S., Ikezawa H., Hayata N., Nakajima R., Morizane C. (2020). Phase 2 study of lenvatinib monotherapy as second-line treatment in unresectable biliary tract cancer: Primary analysis results. BMC Cancer.

[19] Shi C., Li Y., Yang C., Qiao L., Tang L., Zheng Y., Chen X., Qian Y., Yang J., Wu D. (2022). Lenvatinib Plus Programmed Cell Death Protein-1 Inhibitor Beyond First-Line Systemic Therapy in Refractory Advanced Biliary Tract Cancer: A Real-World Retrospective Study in China. Front. Immunol..

[20] Guan R., Zhang N., Deng M., Lin Y., Huang G., Fu Y., Zheng Z., Wei W., Zhong C., Zhao H. (2024). Patients with hepatocellular carcinoma extrahepatic metastases can benefit from hepatic arterial infusion chemotherapy combined with lenvatinib plus programmed death-1 inhibitors. Int. J. Surg..

[21] Zhang W., Zhang K., Liu C., Gao W., Si T., Zou Q., Guo Z., Yang X., Li M., Liu D. (2023). Hepatic arterial infusion chemotherapy combined with anti-PD-1/PD-L1 immunotherapy and molecularly targeted agents for advanced hepatocellular carcinoma: A real world study. Front. Immunol..

[22] Jolissaint J.S., Soares K.C., Seier K.P., Kundra R., Gonen M., Shin P.J., Boerner T., Sigel C., Madupuri R., Vakiani E. (2021). Intrahepatic Cholangiocarcinoma with Lymph Node Metastasis: Treatment-Related Outcomes and the Role of Tumor Genomics in Patient Selection. Clin. Cancer Res..

[23] Yang Z., Fu Y., Wu W., Hu Z., Pan Y., Wang J., Chen J., Hu D., Zhou Z., Chen M. (2023). Comparison of hepatic arterial infusion chemotherapy with mFOLFOX vs. first-line systemic chemotherapy in patients with unresectable intrahepatic cholangiocarcinoma. Front. Pharmacol..

[24] Ishii M., Itano O., Morinaga J., Shirakawa H., Itano S. (2022). Potential efficacy of hepatic arterial infusion chemotherapy using gemcitabine, cisplatin, and 5-fluorouracil for intrahepatic cholangiocarcinoma. PLoS ONE.

[25] Franssen S., Holster J.J., Jolissaint J.S., Nooijen L.E., Cercek A., D’Angelica M.I., Homs M.Y.V., Wei A.C., Balachandran V.P., Drebin J.A. (2024). Gemcitabine with Cisplatin Versus Hepatic Arterial Infusion Pump Chemotherapy for Liver-Confined Unresectable Intrahepatic Cholangiocarcinoma. Ann. Surg. Oncol..

[26] Tabrizian P., Jibara G., Hechtman J.F., Franssen B., Labow D.M., Schwartz M.E., Thung S.N., Sarpel U. (2015). Outcomes following resection of intrahepatic cholangiocarcinoma. HPB.

[27] Burris H.A., Okusaka T., Vogel A., Lee M.A., Takahashi H., Breder V., Blanc J.F., Li J., Bachini M., Zotkiewicz M. (2024). Durvalumab plus gemcitabine and cisplatin in advanced biliary tract cancer (TOPAZ-1): Patient-reported outcomes from a randomised, double-blind, placebo-controlled, phase 3 trial. Lancet Oncol..

[28] Kelley R.K., Ueno M., Yoo C., Finn R.S., Furuse J., Ren Z., Yau T., Klumpen H.J., Chan S.L., Ozaka M. (2023). Pembrolizumab in combination with gemcitabine and cisplatin compared with gemcitabine and cisplatin alone for patients with advanced biliary tract cancer (KEYNOTE-966): A randomised, double-blind, placebo-controlled, phase 3 trial. Lancet.

[29] Kudo M., Finn R.S., Qin S., Han K.H., Ikeda K., Piscaglia F., Baron A., Park J.W., Han G., Jassem J. (2018). Lenvatinib versus sorafenib in first-line treatment of patients with unresectable hepatocellular carcinoma: A randomised phase 3 non-inferiority trial. Lancet.

[30] Jian Z., Fan J., Shi G.-M., Huang X.-Y., Wu D., Liang F., Yang G.-H., Lu J.-C., Chen Y., Ge N.-L. (2021). Lenvatinib plus toripalimab as first-line treatment for advanced intrahepatic cholangiocarcinoma: A single-arm, phase 2 trial. J. Clin. Oncol..

[31] Xie L., Huang J., Wang L., Ren W., Tian H., Hu A., Liang J., Jiao Y., Li Y., Zhou Q. (2022). Lenvatinib Combined with a PD-1 Inhibitor as Effective Therapy for Advanced Intrahepatic Cholangiocarcinoma. Front. Pharmacol..

[32] Chao J., Wang S., Wang H., Zhang N., Wang Y., Yang X., Zhu C., Ning C., Zhang X., Xue J. (2023). Real-world cohort study of PD-1 blockade plus lenvatinib for advanced intrahepatic cholangiocarcinoma: Effectiveness, safety, and biomarker analysis. Cancer Immunol. Immunother..

[33] Auer T.A., Collettini F., Segger L., Pelzer U., Mohr R., Krenzien F., Gebauer B., Geisel D., Hosse C., Schoning W. (2023). Interventional Treatment Strategies in Intrahepatic Cholangiocarcinoma and Perspectives for Combined Hepatocellular-Cholangiocarcinoma. Cancers.

[34] Morawitz J., Bruckmann N.M., Jannusch K., Kirchner J., Antoch G., Loosen S., Luedde T., Roderburg C., Minko P. (2023). Update on Locoregional Therapies for Cholangiocellular Carcinoma. Cancers.

[35] Owen M., Makary M.S., Beal E.W. (2023). Locoregional Therapy for Intrahepatic Cholangiocarcinoma. Cancers.

[36] Amini N., Ejaz A., Spolverato G., Kim Y., Herman J.M., Pawlik T.M. (2014). Temporal trends in liver-directed therapy of patients with intrahepatic cholangiocarcinoma in the United States: A population-based analysis. J. Surg. Oncol..

[37] Hoffmann R.T., Paprottka P.M., Schon A., Bamberg F., Haug A., Durr E.M., Rauch B., Trumm C.T., Jakobs T.F., Helmberger T.K. (2012). Transarterial hepatic yttrium-90 radioembolization in patients with unresectable intrahepatic cholangiocarcinoma: Factors associated with prolonged survival. Cardiovasc. Interv. Radiol..

[38] Ierardi A.M., Angileri S.A., Patella F., Panella S., Lucchina N., Petre E.N., Pinto A., Franceschelli G., Carrafiello G., Cornalba G. (2017). The role of interventional radiology in the treatment of intrahepatic cholangiocarcinoma. Med. Oncol..

[39] Martin R.C.G., Simo K.A., Hansen P., Rocha F., Philips P., McMasters K.M., Tatum C.M., Kelly L.R., Driscoll M., Sharma V.R. (2022). Drug-Eluting Bead, Irinotecan Therapy of Unresectable Intrahepatic Cholangiocarcinoma (DELTIC) with Concomitant Systemic Gemcitabine and Cisplatin. Ann. Surg. Oncol..

[40] Konstantinidis I.T., Groot Koerkamp B., Do R.K., Gonen M., Fong Y., Allen P.J., D’Angelica M.I., Kingham T.P., DeMatteo R.P., Klimstra D.S. (2016). Unresectable intrahepatic cholangiocarcinoma: Systemic plus hepatic arterial infusion chemotherapy is associated with longer survival in comparison with systemic chemotherapy alone. Cancer.

[41] Zhang T., Yang X., Yang X., Zheng K., Wang Y., Wang Y., Sang X., Lu X., Xu Y., Wang X. (2022). Different interventional time of hepatic arterial infusion with PD-1 inhibitor for advanced biliary tract cancer: A multicenter retrospective study. Am. J. Cancer Res..

[42] Wang Y., Xun Z., Yang X., Wang Y., Wang S., Xue J., Zhang N., Yang X., Lu Z., Zhou J. (2023). Local-regional therapy combined with toripalimab and lenvatinib in patients with advanced biliary tract cancer. Am. J. Cancer Res..

[43] Ni J.Y., Sun H.L., Guo G.F., Zhou X., Wei J.X., Xu L.F. (2024). Hepatic arterial infusion of GEMOX plus systemic gemcitabine chemotherapy combined with lenvatinib and PD-1 inhibitor in large unresectable intrahepatic cholangiocarcinoma. Int. Immunopharmacol..

[44] Zitvogel L., Apetoh L., Ghiringhelli F., Kroemer G. (2008). Immunological aspects of cancer chemotherapy. Nat. Rev. Immunol..

[45] Kimura T., Kato Y., Ozawa Y., Kodama K., Ito J., Ichikawa K., Yamada K., Hori Y., Tabata K., Takase K. (2018). Immunomodulatory activity of lenvatinib contributes to antitumor activity in the Hepa1-6 hepatocellular carcinoma model. Cancer Sci..

[46] Yang J., Guo Z., Song M., Pan Q., Zhao J., Huang Y., Han Y., Ouyang D., Yang C., Chen H. (2023). Lenvatinib improves anti-PD-1 therapeutic efficacy by promoting vascular normalization via the NRP-1-PDGFRbeta complex in hepatocellular carcinoma. Front. Immunol..

[47] Jing X., Yang F., Shao C., Wei K., Xie M., Shen H., Shu Y. (2019). Role of hypoxia in cancer therapy by regulating the tumor microenvironment. Mol. Cancer.

